# Retraction: Application of an Effective Statistical Technique for an Accurate and Powerful Mining of Quantitative Trait Loci for Rice Aroma Trait

**DOI:** 10.1371/journal.pone.0193688

**Published:** 2018-03-21

**Authors:** The PLOS ONE Editors

Concerns have been raised regarding redundancy between this PLOS ONE article [1] and other published works by the same authors [2], [3], which were not cited and discussed.

The corresponding author Farahnaz Sadat Golestan Hashemi has stated that the objectives of each study were different: The PLOS ONE article focused on QTL mapping analysis with the inclusion of a cofactor to the analysis [1], the Gene article focused on QTL mapping analysis without the inclusion of any cofactor to the analysis [2], and the Plant Biology article focused on marker assisted selection analysis [3]. Furthermore, Hashemi has stated that the lack of citation was an oversight as a result of the three papers being under review at the same time.

Following evaluation of the issues identified, the PLOS ONE Editors have determined that even if there are small differences in the specific analysis method, the overall approach and main results reported in the PLOS ONE article have been published previously.

The PLOS ONE Editors issue a retraction of the article owing to the concerns about significant redundancy and the lack of disclosure of closely related studies under consideration elsewhere at the time of submission, which is a requirement under the journal’s editorial policies.

The journal editors have notified the authors’ institution. FSGH does not agree with retraction. MYR, MRI, MTMM, HAR, MAL, and FA could not be reached.
